# Ferroptosis: the Good, the Bad and the Ugly

**DOI:** 10.1038/s41422-020-00434-0

**Published:** 2020-11-04

**Authors:** Maceler Aldrovandi, Marcus Conrad

**Affiliations:** 1 Helmholtz Zentrum München, Institute of Metabolism and Cell Death, Ingolstädter Landstr. 1, Neuherberg, 85764 Germany; 2 Laboratory of Experimental Oncology, National Research Medical University, Ostrovityanova 1, Moscow, 117997 Russia

**Keywords:** Cell death, Organelles

**Polyunsaturated ether phospholipids, previously thought to oppose propagation of lipid peroxidation, have been reported to promote the cells’ susceptibility to ferroptotic cell death. A recent study by Zou and colleagues now sheds a new light on the importance of the peroxisome–ether phospholipid axis in modulating ferroptosis susceptibility and evasion**.

**Fig. 1:**
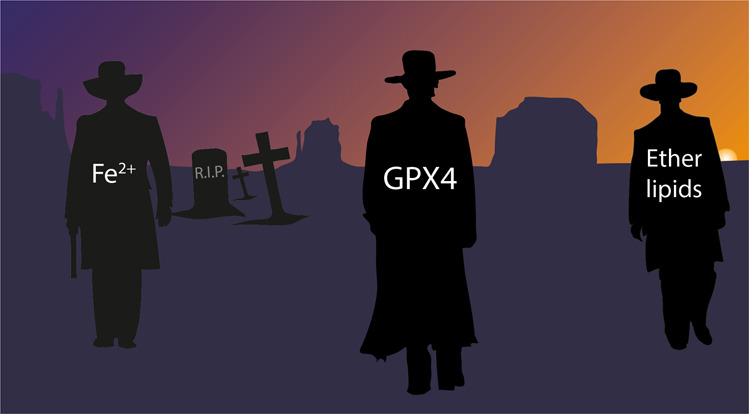
The “Good”, the “Bad” and the “Ugly” dueling in ferroptosis susceptibility and evasion. The two faces of the "Ugly" (i.e., polyunsaturated ePLs) in cell survival and death decisions.

“The Good, the Bad and the Ugly” — an epic Spaghetti Western film directed by Sergio Leone, starring Clint Eastwood, and the iconic soundtrack composed by Ennio Morricone, features a compelling story, in which two men, “the Good” and “the Ugly”, a devious but sympathetic character, form an uncomfortable alliance while looking for treasure. They must also outwit Angel Eyes, “the Bad”, who wants to steal the gold for himself. Ferroptosis, as this classic movie, has some well-known leading players, such as GPX4, “the Good”, iron “the Bad” and ether lipids herein perceived as “the Ugly”, all aiming for “the shining Gold Nugget”, the healthy cell (Fig. 1). While the role of “the Good” and “the Bad” are well established in the ferroptosis field, the true character of “the Ugly” remains mysterious.

Ether phospholipids (ePLs) are an important class of phospholipids characterized by the presence of an alkyl or an alkenyl bond at the *sn-1* position and generally have an ester-linked acyl chain at *sn-2* position of the glycerol backbone. Early studies suggested that ePLs played a noble role. Specifically, enol ether double bonds were deemed as oxidative “sinks”, scavenging a vast variety of oxidants, and therefore protecting nearby polyunsaturated fatty acid (PUFA) acyl chains from oxidation.^1^ In line with this concept, Perez and colleagues have shown that endogenous ether lipids can indeed protect *Caenorhabditis elegans* from dietary dihomo-γ-linolenic acid (DGLA)-induced ferroptosis.^2^ In contrast to these findings, a pro-oxidant effect of PUFA-ePLs has now been reported in the current study by Zou et al.,^3^ whereby the authors focused on elucidating the role of peroxisomes-derived PUFA-ePLs in ferroptotic cell death. Using genome-wide CRISPR/Cas9 suppressor screens, the authors identified several peroxisomal genes, such as *PEX3*, *PEX10* and *PEX12*, which upon depletion, reduced the abundance of peroxisomes. This, in turn, caused a marked decrease in PUFA-ePLs and consequently lowered the sensitivity of human renal and ovarian carcinoma cells to ferroptosis induced by GPX4 inhibition. Similar ferroptosis resistance phenotype and associated changes in PUFA-ePLs were observed following depletion of either peroxisome-associated enzymes alkylglycerone phosphate synthase (AGPS) or fatty acyl-CoA reductase 1 (FAR1), suggesting that the pro-ferroptotic role of peroxisomes is specifically mediated by ether lipid biosynthesis. Interestingly, depletion of the endoplasmic reticulum-resident enzyme 1-acylglycerol-3-phosphate O-acyltransferase 3 (AGPAT3), which selectively incorporates arachidonic acid or docosahexaenoic acid into lysophosphatidic acids, reduced PUFA levels among both ether-linked and diacyl phospholipids, suppressing sensitivity to ferroptosis. Further in vitro studies of diacyl and ether phospholipids confirmed that the PUFA chain, rather than the alkenyl-ether group, was critical for ferroptosis sensitization.

The authors also explored the ability of peroxisomes to promote sensitivity to ferroptosis in vivo. To this end, both ferroptosis-sensitive *GPX4*^*−/−*^ cancer cells and cells additionally depleted of peroxisome (AGPS) or ether lipid biosynthesis genes (*FAR1*, *PEX3* or *PEX10*) were generated and implanted into immunocompromised mice. Co-depleted cancer cells formed larger tumors than parental *GPX4*^*−/−*^ cells, suggesting that the peroxisome–ether lipid axis increases ferroptosis resistance both in vitro and in vivo. Interestingly, in vivo selected ferroptosis-resistant *GPX4*^*−/−*^ cancer cells isolated from these growing tumors led to a robust tumor outgrowth when re-implanted. These cells were not only able to maintain their cell-heritable traits that rendered them insensitive to GPX4 depletion in the first place, but were also capable of self-downregulating PUFA-ePLs, showing a crafty strategy to evade ferroptosis in vivo. Although expression levels of the canonical ferroptosis-modulating genes *ACSL4*, *LPCAT3* and *FSP1* were not significantly altered in these ferroptosis-resistant cells, *AGPS* and *TMEM189* genes were significantly downregulated. However, manipulation of *TMEM189* gene expression alone, which encodes a 1-*O*-alkyl-PE desaturase that converts 1-*O*-alkyl ethers into 1-*O*-alkenyl ethers,^4,5^ did not significantly alter ferroptosis sensitivity, at least in the cell lines tested. This indicates that the pro-ferroptotic role of PUFA-ePLs is independent of the double bond present in the alkenyl ether linkage. By contrast, AGPS inactivation, which depletes both 1-*O*-alkyl-lipids and 1-*O*-alkenyl-lipids, was sufficient to restore growth of *GPX4*^*−/−*^ tumors, suggesting that the spontaneous downregulation of AGPS contributes to the observed emergence of ferroptosis resistance in the carcinoma cell model. Last, the authors explored whether the ferroptosis-sensitizing role of PUFA-ePLs could also be relevant in non-neoplastic settings, specifically in organs with high levels of ePLs. Indeed, differentiated neurons and cardiomyocytes exhibited higher sensitivity to GPX4 inhibition-induced lipid peroxidation and ferroptosis than their progenitor cells, a difference associated with selective upregulation of PUFA-ePLs.

Again, it seems that “the Ugly” has played us all. Although peroxidation of ether lipids has been studied for years, with numerous examples of its antioxidant action demonstrated in cell cultures and in experimental animals, elevated ether lipids might actually represent a yet unrecognized liability. Findings brought to light in this intriguing study indicate that peroxisomes contribute to ferroptosis by synthesizing PUFA-ePLs, which act as substrates for lipid peroxidation that, in turn, increase the cells’ susceptibility to ferroptosis. Vice versa, cells capable of downregulating the peroxisome–ePL axis can evade ferroptotic cell death. The role of endogenous ether lipids in oxidative stress may not be through their general consumption as free radical sinks as previously thought, but rather depend on the intramolecular degree of unsaturation, the chain length of esterified fatty acids and the composition of neighboring lipids as some previous evidences have suggested^6–8^ — this might be perhaps in analogy to what was reported for PUFA-enriched glycerophospholipids.^9,10^ In any case, this is likely not the ending of the story — the plot thickens.
